# From Lipids to Mitochondria: Shared Metabolic Alterations in Obesity and Alzheimer’s Disease

**DOI:** 10.3390/cells15080672

**Published:** 2026-04-10

**Authors:** Romina María Uranga, Shailaja Kesaraju Allani

**Affiliations:** 1 Natural Sciences Division, New College of Florida, 5800 Bay Shore Road, Sarasota, FL 34243, USA; ruranga@ncf.edu; 2 Department of Chemistry and Biochemistry, Charles E. Schmidt College of Science, Florida Atlantic University, Boca Raton, FL 33431, USA; 3 Center for Molecular Biology and Biotechnology, Charles E. Schmidt College of Science, Florida, Atlantic University, Jupiter, FL 33458, USA

**Keywords:** obesity, Alzheimer’s disease, lipids, adipose tissue, mitochondrial dysfunction, TCA cycle, electron transport chain, adipokines, microbiota, gut–brain axis, insulin resistance, glycolysis

## Abstract

**Highlights:**

**What are the main findings?**

- Obesity and Alzheimer’s disease share common metabolic alterations, including adipokine dysregulation, impaired insulin signaling, and mitochondrial dysfunction.
- Mitochondrial dysfunction emerges as a central hub linking systemic metabolic imbalance to neuronal dysfunction.

**What are the implications of the main findings?**

- Early metabolic alterations may precede clinical manifestations, supporting their role as potential biomarkers.
- Targeting mitochondrial function and the gut–brain axis may offer novel strategies to prevent or delay disease progression.

**Abstract:**

The increasing prevalence of obesity and Alzheimer’s disease (AD) in the aging population underscores an urgent need to understand the common cellular and metabolic mechanisms they share. Accumulated evidence suggests that overlapping metabolic disturbances contribute to the pathogenesis of these two conditions. In this review, we highlight key underlying interconnecting metabolic pathways: (1) adipose-brain crosstalk mediated by adipokines and adipose tissue-derived extracellular vesicles that can modulate neuronal function and amyloid pathology, (2) dysregulated lipid metabolism affecting cholesterol, sphingolipids, and phospholipids and thereby promoting inflammation, amyloid precursor protein processing, and tau hyperphosphorylation, (3) impaired glycolysis and insulin resistance, which accelerate both obesity and neurodegenerative processes, (4) mitochondrial dysfunction marked by disrupted tricarboxylic acid cycle enzymes and electron transport chain complexes, leading to elevated reactive oxygen species and driving both obesity and AD pathology, and (5) gut microbiota dysbiosis, which can trigger inflammation as well as amyloid and tau aggregation. Together, these mechanisms show that metabolic alterations appear early, preceding clinical disease, and that understanding these underlying connections can provide strategies to protect metabolic health and prevent disease progression.

## 1. Introduction

The U.S. Census Bureau projects that the number of individuals aged 65 and older will grow significantly over the next few years, reaching 71 million by 2030 and accounting for 20% of the U.S. population [1]. This demographic shift is expected to increase the economic burden on society [2] and, importantly, to elevate the prevalence of age-associated disorders such as Alzheimer’s disease (AD), a neurodegenerative condition characterized by memory impairment and progressive cognitive decline. According to estimates from the Alzheimer’s Society, 1 in 9 (11%) individuals over the age of 65 in the U.S. is diagnosed with AD, making it one of the leading causes of death in the country [3].

At the same time, obesity—a chronic condition characterized by excessive fat accumulation and a major driver of metabolic disorders such as type 2 diabetes, liver disease, and cardiovascular disease—has risen markedly across the lifespan. Notably, its prevalence among older adults nearly doubled from 22% to 40% between 1988 and 2018 [4].

Growing evidence indicates that obesity and AD are mechanistically linked through overlapping metabolic disturbances that contribute to structural and functional alterations in the brain and increase the risk of cognitive decline. These shared disturbances include mitochondrial dysfunction, oxidative stress, and lipid dysregulation, which together disrupt cellular homeostasis and promote neurodegenerative processes.

Given the concurrent rise in both conditions, elucidating their shared metabolic mechanisms has become increasingly important. This review focuses on the interplay between mitochondrial dysfunction, oxidative stress, and lipid dysregulation as converging mechanisms that connect peripheral metabolic imbalance to neurodegeneration [5]. Furthermore, because adipose tissue functions as an endocrine organ, these systemic alterations can influence central nervous system (CNS) function. Accordingly, this review examines how adipose tissue dysfunction influences neurodegeneration, emphasizing the role of metabolic health in shaping cognitive decline.

## 2. Adipose-Brain Crosstalk

As an endocrine organ influencing CNS function, adipose tissue is a key mediator of the complex relationship between metabolic health and cognitive outcomes. Notably, the relationship between adiposity and cognitive decline is not uniform across the lifespan. While obesity in midlife is consistently associated with an increased risk of dementia and AD, this association becomes less clear in older adults. Indeed, lower body weight in late life has been linked to a higher risk of dementia and mortality, a phenomenon often referred to as the “obesity paradox” [6,7,8]. Longitudinal evidence further supports this age-dependent effect: obesity at around 50 years of age is associated with increased dementia risk, whereas obesity at ages 60–70 is not [9]. This discrepancy may be explained by the observation that body mass index (BMI) often begins to decline several years before the clinical onset of dementia. Thus, lower body weight in late life may reflect preclinical neurodegenerative processes rather than a protective metabolic state. Additional factors may also contribute to this apparent contradiction. Aging is accompanied by significant changes in body composition, including loss of muscle mass, redistribution of fat, and reductions in height, all of which limit the accuracy of BMI as a surrogate marker of adiposity in older individuals. In addition, unintentional weight loss—frequently observed in the elderly—is strongly associated with increased morbidity and mortality and often reflects underlying subclinical disease. These observations underscore the importance of assessing adipose tissue function, rather than relying solely on anthropometric measures such as BMI, to understand the links between metabolic health and cognitive outcomes.

Importantly, beyond age-related effects, variability in metabolic health among individuals with obesity adds further complexity. Excess adiposity does not uniformly translate into metabolic dysfunction. A substantial subset of individuals with obesity—estimated to range broadly from 2% to 50% depending on the population and diagnostic criteria—exhibits a relatively preserved metabolic profile. These individuals lack the classical features of insulin resistance, dyslipidemia, or hypertension [10,11,12]. This phenotype, commonly referred to as metabolically healthy obesity (MHO), remains a matter of debate. Although initially considered benign, longitudinal studies indicate that these individuals may still carry an elevated cardiovascular risk compared to metabolically healthy lean counterparts [13].

Understanding these discrepancies requires a closer examination of adipose tissue biology. Adipose tissue is a highly dynamic and heterogeneous organ composed of a specialized cellular network, including adipocytes, pre-adipocytes, immune cells, and endothelial cells, collectively termed the stromal vascular fraction. Beyond its role as an energy reservoir, in humans, adipose tissue is classified into two main anatomical and physiological depots: subcutaneous adipose tissue (SAT) and visceral adipose tissue (VAT). These depots differ significantly not only in their location but also in their developmental origin, expansion patterns, and metabolic profiles. While SAT primarily expands through hyperplasia (increase in cell number) and acts as a metabolic buffer by storing excess lipids, VAT tends to expand via hypertrophy (increase in cell size). Hypertrophic VAT is highly pro-inflammatory and exhibits a detrimental secretory profile, releasing free fatty acids and inflammatory cytokines directly into the portal circulation, which contrasts with the more protective or neutral profile of SAT.

**Figure 1:**
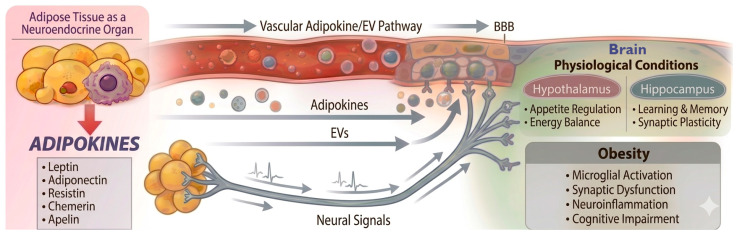
Adipose–brain crosstalk. Adipose tissue acts as a neuroendocrine organ, modulating brain function through three primary routes: the systemic secretion of adipokines and extracellular vesicles (EVs) that cross the blood–brain barrier (BBB), and direct neural signals. Under physiological conditions, these signals maintain neuronal homeostasis. Dysregulation during obesity alters this communication, promoting neuroinflammation and microglial activation, which contribute to neurodegenerative processes and cognitive impairment.

As a true neuroendocrine organ, adipose tissue regulates systemic metabolism through a continuous bidirectional communication with the CNS. This crosstalk integrates multiple types of signals, including adipokines, neural pathways, and extracellular vesicles (EVs). Together, these signals coordinate processes such as energy homeostasis, insulin sensitivity, systemic inflammation, and brain function. Disruptions in adipose-brain communication—driven primarily by altered adipokine and EV secretory patterns under conditions such as obesity and metabolic syndrome—contribute to neuroinflammation, neuronal dysfunction, and cognitive decline [14,15] (Figure 1).

### 2.1. Adipokines

As mentioned above, one of the key mechanisms through which adipose tissue communicates with both peripheral organs and the brain is via adipokines. Adipokines, also known as adipo(cyto)kines, are bioactive molecules primarily secreted by adipocytes that play key roles in the regulation of energy metabolism, inflammation, immunity, and cardiovascular function, among other processes [16]. However, macrophages infiltrating adipose tissue also contribute significantly to adipokine secretion, thereby integrating systemic metabolism with the immune system [17,18].

Like classical cytokines, adipokines play a relevant role in inflammatory processes. They include pro-inflammatory mediators such as leptin, chemerin, resistin, adipsin, TNF-α, IL-6, and PAI-1; anti-inflammatory factors such as adiponectin, omentin, vaspin, and TGF-β1; and adipokines with dual, context-dependent, or at least controversial, functions, such as visfatin [19,20]. What ultimately matters, however, is not the effect of each individual adipokine, but rather the overall adipokine profile secreted by adipose tissue and its systemic impact. Under physiological conditions, a balance exists between pro- and anti-inflammatory signals; however, in obesity, this balance is disrupted, shifting toward a chronic, low-grade pro-inflammatory state. This altered secretory pattern contributes not only to the metabolic disturbances associated with obesity but also exerts central effects, promoting neuroinflammation and even impairing brain health [18,21,22].

#### 2.1.1. Adipokines and the Brain: Transport, Function, and Controversies

**Figure 2:**
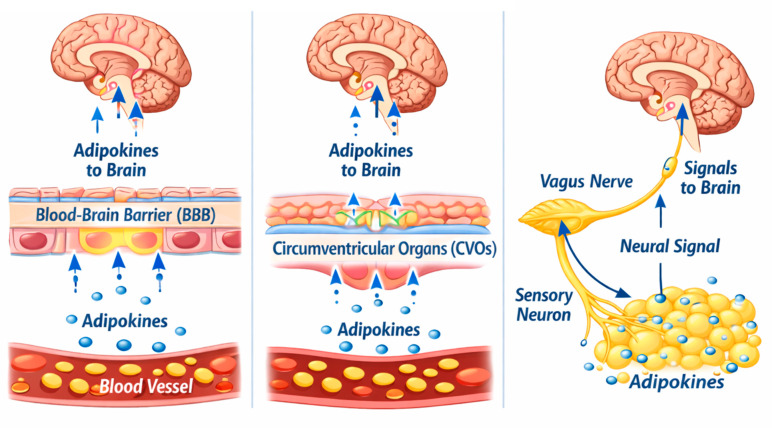
Pathways of adipokine signaling from the periphery to the brain. Adipokines, secreted by adipose tissue, can reach the brain through multiple routes: via the blood–brain barrier (BBB), where adipokines cross endothelial cells to act directly on brain regions (**left panel**); via the circumventricular organs (CVOs), specialized brain structures lacking a complete BBB, allowing adipokines in the blood to interact with neural tissue (**middle panel**); and via the vagus nerve, in which adipokines act on sensory neurons in adipose tissue to generate neural signals that are transmitted to the brain (**right panel**).

Although the peripheral metabolic role of adipokines has been well established, many aspects of their actions in the brain remain under debate. Some adipokines are known to reach the CNS, either by crossing the blood–brain barrier (BBB) or via circumventricular regions. However, controversies persist regarding the exact mechanisms, the identity of the transporter, and whether crossing the BBB is required for specific central effects. For example, leptin, one of the best characterized adipokines, is a 16 kDa peptide hormone released into the circulation by white adipose tissue (WAT) in proportion to total body fat, and it maintains energy homeostasis by acting on hypothalamic neurons [23]. Leptin can reach the brain either by crossing the BBB or via circumventricular regions. In addition, afferent sensory pathways innervating WAT and transmitting signals to the brain are known to be stimulated by leptin, thereby providing another mechanism through which leptin conveys information to the brain [24] (Figure 2). Nevertheless, it remains unclear which central effects of leptin require its passage across the BBB. Also, both the mechanisms of transport and the identity of the transporter remain a matter of debate [25,26,27]. Moreover, leptin production within the brain has been reported in both humans and rodents [28,29,30,31].

In the case of other adipokines found in the brain, such as adiponectin, which is the most abundant in plasma and plays an important role in regulating glucose and lipid metabolism, there is no evidence of cerebral production, and for a long time it was thought not to cross the BBB [32]. However, its detection in the cerebrospinal fluid (CSF) suggests that it does reach the CNS, although the exact mechanism has not been fully defined [33,34,35].

Despite these mechanistic controversies, the accumulated evidence is consistent: signals originating from adipose tissue reach the brain, modulate both neurons and glial cells, and play a significant role in the development of neuroinflammatory and neurodegenerative processes, which is particularly relevant in the context of AD.

#### 2.1.2. Adipokines, Neuroinflammation, and AD

As aforementioned, these adipose-derived signals represent one of several factors that may influence neuroinflammation, a process increasingly recognized as a central component in the progression of neuronal dysfunction in AD. Chronic systemic inflammation associated with obesity can directly affect the brain by impairing BBB integrity. This increased permeability allows pro-inflammatory molecules and immune cells to enter the brain parenchyma. This process promotes sustained activation of microglia and astrocytes, the establishment of a neuroinflammatory milieu, alterations in synaptic plasticity, neuronal dysfunction, and cognitive decline [36].

Within this framework, adipokines emerge as the primary messengers linking metabolic status to neurodegeneration. However, the evidence supporting their role in AD is characterized by significant translational gaps between preclinical models and human clinical data, as well as inconsistencies among human studies. This variability is already evident at the preclinical level: mechanistic in vitro studies exhibit substantial variations depending on the use of immortalized neuronal cell lines or primary neuronal cultures derived from distinct brain regions. Furthermore, the lack of standardized adipokine concentrations and variable exposure times across experimental designs hampers reproducibility.

These complexities are further compounded in animal models. The use of diverse species and various transgenic strains—such as those modeling amyloid-β peptide (Aβ) or tau pathology—adds layers of difficulty to the analysis. Additionally, the frequent omission of sex as a fundamental variable, or the exclusive use of single-sex cohorts, often masks dimorphic responses. Such differences are essential for understanding a disease that disproportionately affects females. In human studies, the challenge intensifies due to the lack of uniformity in cohort composition across age, sex, and ethnicity, as well as the complexity of accurately distinguishing between mild cognitive impairment (MCI), different forms of dementia, and AD, despite advances in biomarker-based diagnostics.

A prominent example of this disconnect is observed in the study of adiponectin and leptin. In preclinical models, both adipokines exhibit neuroprotective effects, including reduced Aβ production, enhanced Aβ degradation, and decreased tau phosphorylation [37,38,39,40,41,42]. Paradoxically, these findings often fail to translate into human cohorts [43]. Clinical studies have reported higher serum adiponectin levels in AD patients compared to those with MCI, and these levels have even been shown to predict faster rates of cortical thinning [44,45]. This discrepancy may be explained by several factors that limit the strength of current evidence. First, most clinical assays do not differentiate between adiponectin isoforms (low- vs. high-molecular-weight forms), which have distinct biological activities. Second, elevated adiponectin levels in AD may reflect a compensatory but ineffective response to systemic metabolic stress or “adiponectin resistance” [46]. Similarly, although leptin inhibits Aβ fibrillogenesis in vitro, its protective effects in humans are often masked by leptin resistance, particularly in obese individuals.

Beyond leptin and adiponectin, other less extensively studied adipokines, such as apelin, visfatin, and chemerin, have shown promising benefits in diabetes-related cognitive impairment [47,48,49,50,51]. Nevertheless, this evidence remains largely restricted to animal models and lacks large-scale human validation. The field also faces significant technical variability in adipokine determination, with results differing based on the sample type—be it serum, plasma, whole blood, or CSF—and the sensitivity of the assays used. Finally, the fact that both deficiency and excess of many adipokines can be detrimental further complicates defining a “normal” clinical range and drawing definite conclusions. Consequently, future research must shift toward standardized investigations that account for adipose tissue distribution (visceral vs. subcutaneous) and specific adipokine “signatures” to establish reliable biomarkers for AD progression and potential therapeutic targets.

### 2.2. Adipose Tissue-Derived Small EVs

Beyond soluble mediators such as adipokines, adipose tissue also communicates through EV-based mechanisms. EVs are nanosized structures enclosed by lipid bilayers that are naturally released by a wide variety of cells, including multiple cell types within adipose tissue. Based on their size and biogenesis, EVs are commonly classified into exosomes, microvesicles, and apoptotic bodies [52]. Adipose tissue has emerged as a major source of EVs. These EVs mediate intercellular communication within adipose tissue and also serve as key messengers in interorgan communication between adipose tissue and distant organs such as the brain, heart, liver, and immune system [53]. These vesicles transport a wide range of bioactive cargo, including lipids, mRNA (which can be translated into protein in recipient cells), various classes of non-coding RNAs (such as lncRNAs, miRNAs, and circRNAs), proteins, DNA, and metabolites. Notably, recent studies have revealed a remarkable EV-mediated exchange of mitochondrial components, and even whole mitochondria, between adipose tissue cells as well as between adipose tissue and other organs, highlighting an additional layer of metabolic and functional crosstalk [54,55,56,57].

#### Adipose Tissue-Derived EVs in the Brain

As previously noted, the adipokine secretory profile of adipose tissue is well characterized in obesity. In contrast, changes in the cargo of EVs have been less extensively studied, and their effects on the brain remain an emerging area of investigation. Notably, some studies have shown that adipose tissue-derived EVs can cross the intact BBB, with EVs from VAT appearing to target the brain more efficiently than those from SAT or brown adipose tissues [58]. While these findings are intriguing, further research is needed to establish their physiological relevance and causal roles in brain function.

The effects of adipose tissue EVs on the hypothalamus have been demonstrated, specifically targeting proopiomelanocortin (POMC) neurons in mice. These EVs produce differential effects on appetite and body weight depending on their source. For example, EVs from obese mice, when administered to lean mice, stimulate appetite and promote weight gain, whereas EVs from lean mice, when introduced into mice fed a high-fat diet (HFD), suppress appetite and induce weight loss [59].

Given the strong association of obesity and metabolic syndrome with AD, researchers have also begun exploring the role of adipose tissue-derived EVs in the hippocampus, a brain region known to be critically involved in learning and memory and one of the earliest affected in AD pathology. Alterations in hippocampal structure and function are thought to contribute to cognitive decline in AD, making this region a key focus for understanding disease mechanisms. Interestingly, in vivo experiments in which an adeno-associated virus (AAV) expressing a fluorescent EV reporter was injected into mouse adipose tissue revealed the marker in the brain two weeks later, with marked enrichment in the hippocampus and colocalization with neuronal populations. In parallel, EVs from HFD-fed mice, labeled with a fluorescent dye and injected intravenously, were detected in the hippocampus of chow-fed control animals, where they exhibited a preferential association with neurons over glial cells [58]. These findings indicate that adipose tissue-derived EVs can reach the brain under conditions in which the BBB remains structurally intact, although the precise mechanisms mediating their entry into the CNS have not been addressed in this study. Notably, EVs have been reported to traverse multiple brain barriers, including the BBB, the blood–CSF barrier, and the ependyma [60]. While the molecular mechanisms underlying EV transport across these interfaces remain incompletely defined, evidence suggests the involvement of active processes such as receptor-mediated transcytosis and other endocytic pathways. In the same study by Wang et al. (2022), it was further shown that specifically adipose tissue EVs (not liver-derived EVs) are able to reach the brain, and that miRNAs carried within these vesicles, derived from either HFD-fed animals or patients with diabetes mellitus, can promote synaptic loss and cognitive decline in recipient animals [58]. These results point to a potential mechanistic link between peripheral metabolic dysfunction and obesity-associated cognitive impairment, although further research is needed to elucidate the precise pathways involved.

In addition to animal studies, human data also support the role of adipose tissue EVs in modulating neuronal functions. Another study reported that FABP4+ EVs (suggestive of adipose origin, although not conclusive) from CSF and plasma of AD patients were enriched in miRNAs predicted to reduce neuronal plasticity [57,61]. These findings suggest a significant role for adipose tissue EVs in potentially linking metabolic status to cognitive function.

A recent study sought to investigate the link between the content of adipose tissue EVs and AD pathogenesis more directly. The researchers isolated EVs from VAT and SAT of both obese and lean individuals and analyzed their lipid profiles. Subsequently, they assessed the effects of these lipid profiles on the fibrillization of human Aβ peptides 1-40 and 1-42. EVs from obese individuals were found to promote Aβ aggregation, with lysophosphatidylcholine and sphingomyelin from these EVs specifically dysregulating Aβ fibrillization [62].

Although numerous studies have investigated the impact of adipose tissue EVs on other organs, their role in neurodegenerative diseases such as AD remains largely unexplored. Only a handful of studies, cited here, have addressed this question, highlighting the need for additional research with clinical and translational relevance.

Collectively, accumulating evidence indicates that adipose tissue plays an active role in the regulation of brain physiology through multiple communication pathways, including adipokines and EVs. In obesity, alterations in the adipose secretory profile promote a chronic pro-inflammatory environment that can affect the CNS, contributing to neuroinflammation, neuronal dysfunction, and cognitive decline. Emerging data suggest that adipose-derived EVs may directly influence neuronal signaling and amyloid pathology, providing new mechanistic links between peripheral metabolic dysfunction and neurodegenerative processes such as AD. Although these findings highlight adipose tissue as a potential contributor to brain pathophysiology and a promising therapeutic target, further studies are required to clarify the precise molecular mechanisms connecting adipose dysfunction with neurodegeneration.

## 3. Lipid Metabolism and the Brain

While adipokines and adipose-derived EVs mediate significant communication between adipose tissue and the CNS, obesity also profoundly alters systemic and cerebral lipid metabolism. This provides an additional metabolic axis through which adipose dysfunction can influence brain health. Indeed, among the mechanisms linking peripheral metabolic dysfunction to neuronal perturbations, elevated circulating free fatty acids and altered lipid handling play a central role. These changes can perturb neuronal membrane composition, myelination, and lipid-dependent signaling pathways. These metabolic disruptions can synergize with neuroinflammatory and EV-mediated mechanisms, further promoting neuronal dysfunction and vulnerability to neurodegenerative processes.

These systemic lipid alterations originate from the expansion and active remodeling of WAT in obesity, which involves adipocyte hypertrophy, insulin resistance, and macrophage infiltration. At the same time, macrophages shift from an anti-inflammatory to a pro-inflammatory phenotype, all of which result in dysfunctional adipose tissue [63]. Insulin-resistant adipocytes exhibit increased lipolysis, leading to elevated circulating free fatty acids that promote lipotoxicity in non-adipose tissues such as the liver and skeletal muscle, which in turn exacerbates insulin resistance in these organs.

Importantly, insulin resistance develops earlier in muscle than in the liver. As glucose uptake in muscle declines, excess glucose is redirected to the liver, stimulating de novo lipogenesis and further exacerbating hyperlipidemia [64]. This systemic metabolic imbalance highlights the role of adipose tissue as a lipid sink that buffers excess lipids and protects against ectopic fat deposition. When this lipid-buffering capacity is exceeded, lipids accumulate in non-adipose organs, a phenomenon strongly associated with insulin resistance, impaired glucose and lipid metabolism, and increased cardiovascular risk.

Obesity-driven alterations in lipid handling give rise to metabolic syndrome. Metabolic syndrome is defined by the presence of at least three of the following features: abdominal obesity, hypertriglyceridemia, low plasma high-density lipoprotein (HDL) cholesterol, hypertension, and elevated blood glucose. Obesity-associated dyslipidemia is characterized by elevated plasma levels of triglycerides, free fatty acids, very-low-density lipoprotein (VLDL), and non-HDL cholesterol. It also includes reduced plasma HDL cholesterol and qualitative alterations in low-density lipoprotein (LDL) and HDL particles [65]. In clinical practice, hypertriglyceridemia and/or low HDL cholesterol levels are the primary diagnostic criteria.

Dysregulated lipid metabolism in obesity contributes to brain dysfunction. Although the systemic effects of dyslipidemia have long been recognized, its link to AD has been intensively studied only in recent decades. Alterations in brain lipid metabolism are associated with the onset and progression of neurodegeneration, positioning cerebral lipids as potential therapeutic targets. Moreover, because CSF contains metabolites that diffuse from the brain and reflect cerebral metabolic status, CSF lipid profiles are being explored as early molecular biomarkers of neurodegenerative disease.

Among lipid classes, alterations in brain cholesterol metabolism and distribution are strongly associated with AD. Apolipoprotein E (APOE) is the primary cholesterol transporter in the brain. The APOEε4 allele represents a major genetic risk factor for AD. Although a direct causal relationship between cholesterol levels and amyloidogenesis has not been conclusively established, cholesterol-enriched lipid rafts modulate amyloid precursor protein (APP) processing and the activity of key enzymes such as β-site APP cleaving enzyme 1 (BACE1) and γ-secretase. The sequential actions of these enzymes give rise to Aβ peptides of different lengths, with Aβ1-40 and Aβ1-42 representing the major species involved in neuritic plaque aggregation [66]. These observations suggest an indirect but functionally relevant link between cholesterol metabolism and AD pathogenesis [67,68,69].

In addition to cholesterol, sphingolipids have been extensively studied in the context of AD, likely due to their close association with lipid rafts. Brains from AD patients show reduced sphingomyelin and increased ceramide levels. This is largely driven by elevated acid sphingomyelinase activity. Ceramides stabilize BACE1, thereby promoting Aβ generation from APP. Furthermore, GM1 gangliosides have been shown to sequester Aβ peptides on neuronal membranes, disrupting neuronal structure and function [70,71,72].

Importantly, brain cholesterol homeostasis is largely independent of peripheral cholesterol pools. The BBB prevents the free exchange of circulating cholesterol and lipoproteins [73,74]. Instead, most brain cholesterol is synthesized locally, primarily by astrocytes, and redistributed to neurons via APOE-containing lipoproteins [75]. Nevertheless, this paradigm is not absolute, and some degree of crosstalk between peripheral and brain cholesterol metabolism has been proposed. In particular, cholesterol-derived metabolites such as oxysterols can cross the BBB and contribute to the regulation of cholesterol homeostasis in both compartments [73]. Moreover, experimental evidence suggests that small amounts of peripheral cholesterol may enter the brain under specific conditions, such as hypercholesterolemia or BBB dysfunction. Therefore, while brain and peripheral cholesterol pools are largely segregated, their metabolic interplay remains an area of active investigation, adding complexity to the interpretation of cholesterol’s role in AD pathogenesis.

Phospholipids also play a critical role in AD pathogenesis. They strongly influence γ-secretase activity and APP processing. Alterations in the metabolism of phosphatidylcholine (PC), phosphatidylethanolamine (PE), and phosphatidylinositol (PI), as well as changes in phospholipase C (PLC) and phospholipase D (PLD) activity, have been reported in both experimental models and AD patients [69,70,71,72,76]. Furthermore, Bazan et al. (2005, 2009) [77,78] demonstrated that docosahexaenoic acid (DHA)-derived mediators such as neuroprotectin D1 (NPD1) promote neuronal survival and reduce Aβ production and neurotoxicity. This highlights a neuroprotective mechanism mediated by lipid-derived molecules. Importantly, dysregulated phospholipid metabolism affects multiple AD-related mechanisms [77,78]. First, it alters plasma membrane fluidity and permeability, directly influencing amyloidogenic enzyme activity and Aβ deposition. Second, it generates lipid-derived metabolites that act as mediators of neuroinflammation. These mediators promote microglial activation and sustained production of pro-inflammatory cytokines [79,80].

Consistent with these observations, a recent spatiotemporal lipidomic study provided further insight into lipid metabolic dysregulation in AD mouse models, particularly affecting sphingolipid and glycerophospholipid pathways [80]. The authors identified imbalances in enzymes involved in diacylglycerol (DAG) metabolism, including PLC, leading to early DAG accumulation. These findings are in agreement with previous lipidomic analyses of AD patient samples. Notably, lipid alterations were not confined to the hippocampus, a region highly vulnerable to AD, but were also detected in the cortex and thalamus, with the most pronounced changes observed in the thalamus. Moreover, amyloid plaque deposition, neurodegeneration, and microglial activation correlated spatially with the distribution of lipid metabolic changes.

Together, these findings highlight the important contribution of lipid metabolism in AD pathogenesis. However, the cellular specificity of these alterations remains insufficiently characterized beyond regional differences. Furthermore, while several lipid species are being proposed as early biomarkers of AD, future studies must establish robust correlations between lipid alterations in the brain and those detected in less invasive samples such as blood or CSF. Although dysregulated lipid metabolism clearly influences AD progression, the direct causal links between these alterations and amyloid pathology remain incompletely defined, and most evidence comes from in vitro or animal models. In obesity, adipose tissue dysfunction disrupts systemic lipid metabolism, promoting dyslipidemia and ectopic lipid deposition, which in turn may impact the brain. Thus, lipid metabolism emerges as a critical mechanistic link between obesity and neurodegeneration.

## 4. Glycolysis and Insulin Sensitivity

In addition to lipid dysregulation, obesity-driven metabolic disturbances profoundly impact glucose handling and insulin signaling in the brain. These disturbances affect the pathways by which neurons generate energy from glucose. Glycolysis is a central process in this context. It is a cytoplasmic pathway that breaks down glucose into two molecules of pyruvate, producing ATP and NADH while supplying intermediates for other metabolic pathways. When oxygen is available, pyruvate enters the mitochondria and is converted into acetyl-CoA, fueling the tricarboxylic acid (TCA) cycle, which is followed by the electron transport chain (ETC) to generate additional ATP. Under anaerobic conditions, pyruvate is reduced to lactate, thereby regenerating NAD^+^ and allowing glycolysis to continue [81].

Glucose is the primary energy source for the brain. It is crucial for ATP production and neurotransmitter synthesis. In patients with sporadic AD, cerebral glucose metabolism is reduced, ATP levels decline, and insulin signaling is impaired [82]. These conditions promote intracellular accumulation of Aβ and tau hyperphosphorylation. Restoring brain glucose metabolism could therefore represent a potential therapeutic strategy in AD [83].

Insulin resistance is a major contributor to altered glucose metabolism. Insulin, secreted by pancreatic β-cells, regulates glucose uptake, storage, and utilization in peripheral tissues. Activation of the insulin receptor triggers the PI3K/Akt pathway, promoting GLUT4 translocation and glucose uptake. However, in the brain, glucose entry is largely insulin-independent and occurs through GLUT1 or GLUT3 transporters. In this context, insulin is thought to primarily regulate intracellular signaling and metabolic pathways in neurons and glial cells [84]. Impairments in brain insulin signaling have been observed decades before AD develops, contributing to disease progression by impairing energy metabolism, synaptic plasticity, and Aβ clearance. Consequently, AD has been referred to as “type 3 diabetes.” Animal studies using intracerebral streptozotocin demonstrate that inhibition of β-cell insulin production induces early AD-like features, including memory deficits, which can be partially reversed by antidiabetic drugs [85].

Brain insulin resistance also contributes to oxidative stress, mitochondrial dysfunction, energy deficits, abnormal tau phosphorylation, and neuroinflammation [86]. However, it is not sufficient on its own to fully cause AD. Rather, AD represents a selective neuroendocrine disorder in which impairments in insulin and insulin-like growth factor signaling contribute to, but do not solely drive, the molecular and histopathological hallmarks of the disease [86].

While these mechanisms are critical in the brain, obesity also affects peripheral tissues, including the liver and muscle, further contributing to systemic metabolic dysfunction that may exacerbate neurodegenerative processes. Glycolytic enzymes such as hexokinase, phosphofructokinase, and pyruvate kinase are upregulated in obese mice and patients [87,88], suggesting enhanced glucose metabolism under obesogenic conditions. Insulin also regulates fatty acid oxidation and mitochondrial function, and insulin resistance impairs these processes in muscle and adipose tissue, limiting mitochondrial oxidative metabolism [89,90,91].

Excess lipid accumulation due to impaired fatty acid oxidation promotes mitochondrial stress. In hepatocytes from obese mice, reverse electron transport at coenzyme Q in complex I generates excessive reactive oxygen species (ROS) and disrupts mitochondrial metabolism [92]. This oxidative stress contributes to progressive cognitive decline and may exacerbate AD pathology [93].

Overall, disruptions in glycolysis and insulin signaling compromise neuronal energy availability. These perturbations stress mitochondria, impair oxidative phosphorylation, increase ROS production, and contribute to neurodegeneration. Thus, central and peripheral metabolic dysregulation, together with mitochondrial vulnerability, are intimately linked in the context of AD.

## 5. Mitochondrial Dysfunction and Oxidative Stress

Mitochondria are double-membraned, endosymbiotic organelles essential for cellular energy metabolism. Beyond ATP production, they regulate lipid metabolism, calcium homeostasis, redox signaling, and apoptosis. Dysfunctional mitochondria generate less ATP, produce excessive ROS, and disrupt metabolic programming, thereby contributing to disease [94].

Although the brain accounts for approximately 2% of body weight, it consumes nearly 20% of total energy, reflecting neurons’ high metabolic demands [95]. Impaired energy supply compromises synaptic transmission and neuronal survival [96]. Mitochondrial dysfunction is proposed as a primary driver in sporadic, late-onset AD, contributing to both Aβ plaque and neurofibrillary tangle formation. The mitochondrial cascade hypothesis, first proposed by Swerdlow and Khan (2004), provides a unifying framework for AD pathology, suggesting that mitochondrial dysfunction precedes Aβ deposition, tau phosphorylation, and neurodegeneration [97,98]. Aging and gene-environment interactions exacerbate this decline. This view is consistent with the free radical theory of aging, which proposes that ETC activity declines with age while mitochondrial-derived oxidative stress increases. Once a critical threshold is reached, synaptic failure and neuronal death ensue [99].

Evidence from AD models and patient studies shows reduced ATP levels, impaired respiratory chain function, and excessive ROS generation [99,100]. Disrupted mitochondrial dynamics, including fission and fusion, further compromise bioenergetic stability and neuronal function [101].

Obesity also involves mitochondrial dysfunction. Excess nutrient intake alters fatty acid oxidation, impairs the ETC, increases ROS production, and reduces mitochondrial efficiency in adipocytes [102,103]. High-fat or high-sucrose diets promote inflammation, oxidative stress, mitochondrial aging, and impaired fusion, leading to Aβ accumulation and cognitive decline [104]. These findings emphasize a mechanistic link between obesity, mitochondrial impairment, and neurodegeneration [105].

**Figure 3:**
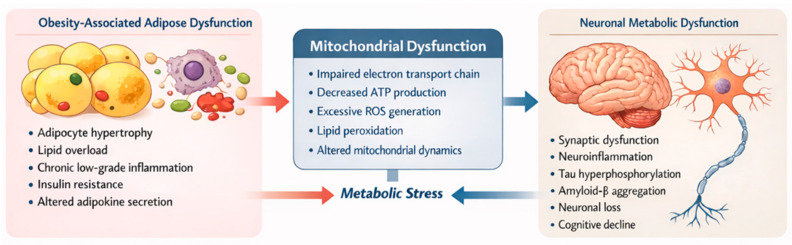
Mitochondrial dysfunction as a central metabolic hub. Chronic low-grade inflammation, insulin resistance, and altered adipokine secretion from hypertrophic adipocytes trigger systemic metabolic stress. This stress drives mitochondrial dysfunction, characterized by impaired electron transport, reduced ATP production, and excessive ROS generation. Consequently, these central metabolic defects promote neuroinflammation, synaptic dysfunction, and the accumulation of tau and amyloid-β aggregates, ultimately leading to neuronal loss and cognitive decline.

Together, these observations suggest that mitochondrial dysfunction represents a common mechanism linking obesity and AD (Figure 3). In the following sections, we will explore how alterations in the TCA cycle, the ETC, energy production, and oxidative stress contribute to both neuronal and systemic metabolic deficits.

### 5.1. TCA Cycle in AD and Obesity

The TCA cycle plays a central role in mitochondrial energy metabolism by integrating carbohydrate, lipid, and amino acid oxidation to sustain ATP production. Given the high energetic demand of the brain, even subtle disruptions in the TCA cycle function can compromise neuronal activity and survival. In both AD and obesity, accumulating evidence indicates that TCA cycle alterations represent a shared feature of metabolic dysfunction, ultimately contributing to impaired bioenergetics and disease progression [106,107,108].

Rather than affecting a single enzymatic step, both conditions are characterized by coordinated alterations across multiple nodes of the TCA cycle, reflecting a broader mitochondrial reprogramming. One prominent pattern involves impaired entry and flux through the cycle, as indicated by reduced citrate synthase activity in both AD and obesity, which limits acetyl-CoA utilization, ATP production, and biosynthetic pathways such as acetylcholine synthesis [109,110,111,112,113,114,115]. This restriction at the entry point is further compounded by alterations in downstream enzymes, collectively reducing cycle efficiency. As summarized in Supplementary Table S1, these enzyme alterations are context-dependent, varying across tissues and disease stages, but consistently converge on disrupted cellular energetics.

A second recurring feature is the disruption of redox balance and metabolic coupling, particularly involving enzymes such as aconitase and isocitrate dehydrogenase (IDH), which are highly sensitive to oxidative stress. Decreased IDH activity in AD and obesity is associated with reduced NADH and NADPH production, compromising both energy generation and antioxidant defenses [116,117,118,119,120,121]. Similarly, the susceptibility of aconitase to oxidative inactivation links TCA dysfunction directly to increased ROS, reinforcing a cycle of mitochondrial damage.

Another key pattern is the impairment of critical regulatory nodes that integrate metabolism and signaling, such as the α-ketoglutarate (α-KG) dehydrogenase complex. Reduced α-KG levels and enzyme activity have been associated with cognitive decline in AD and altered adipogenesis and metabolic regulation in obesity, highlighting a convergence between neuronal and systemic metabolic dysfunction [117,122,123]. In parallel, alterations in succinate metabolism further illustrate this integrative role, as succinate acts not only as a metabolic intermediate but also as a signaling molecule influencing inflammation, thermogenesis, and epigenetic regulation [124,125,126,127,128].

Finally, late-stage TCA cycle enzymes, including fumarase and malate dehydrogenase, exhibit differential activity in AD and obesity. In AD, mixed fumarase activity might reflect a stage-dependent compensatory response, while elevated malate dehydrogenase in CSF could represent mitochondrial structural failure [117,123,129]. In obesity, the fumarate-to-succinate flux drives oxidative stress while a deficiency in fumarase provides protection [130,131]. These changes are often tissue- and stage-dependent but consistently point toward disrupted NADH production, altered redox state, and impaired regeneration of oxaloacetate, further limiting cycle continuity [132,133,134,135].

Importantly, in the context of obesity, chronic nutrient excess—particularly elevated free fatty acids—can overload the TCA cycle, leading to incomplete oxidation, accumulation of intermediates, and enhanced ROS production. In AD, similar bioenergetic failure emerges in the setting of glucose hypometabolism, suggesting that distinct upstream triggers converge on a common mitochondrial phenotype.

Collectively, these findings indicate that AD and obesity share a pattern of TCA cycle dysfunction characterized by impaired flux, redox imbalance, and metabolic inflexibility, ultimately contributing to mitochondrial dysfunction and energy deficits. This supports the concept that alterations in central carbon metabolism are not merely secondary consequences but active drivers of neurodegenerative and metabolic disease processes.

### 5.2. ETC in AD and Obesity

The mitochondrial ETC is the primary site of oxidative phosphorylation, coupling electron transfer to ATP production [136]. As observed for the TCA cycle, in both AD and obesity, growing evidence indicates that alterations in ETC function are not restricted to individual complexes but instead reflect system-wide disruptions that impair energy production and increase ROS generation.

A central feature of ETC dysfunction in both conditions is an imbalance between electron transport and oxidative phosphorylation efficiency. Reductions in the activity of multiple complexes—particularly complexes I, IV, and V—have been consistently reported in AD, leading to decreased ATP production and impaired neuronal function [99,137,138,139,140,141]. Similar impairments are observed in metabolic disorders, where reduced ETC efficiency limits energy availability in high-demand tissues such as the brain and skeletal muscle [94,142,143,144,145]. Importantly, these alterations often appear early in disease progression, supporting the idea that mitochondrial dysfunction is an initiating rather than secondary event.

Another hallmark is the dysregulation of mitochondrial ROS production, primarily originating from complexes I and III [146,147,148]. Under physiological conditions, ROS act as signaling molecules; however, in AD and obesity, excessive ROS generation overwhelms antioxidant defenses, causing oxidative damage to mitochondrial DNA, proteins, and lipids. Interestingly, the role of ROS is highly context-dependent: while elevated ROS contributes to neurodegeneration in AD, controlled ROS production in adipose tissue is required for processes such as adipocyte differentiation [149]. This dual role highlights the fine balance between physiological signaling and pathological damage.

ETC dysfunction is also marked by compensatory and context-dependent responses. For example, complex I activity may decrease in association with tau pathology in AD, yet increase in certain peripheral tissues or experimental conditions, suggesting adaptive responses to fluctuating energy demands [137,150]. Similarly, HFD models show upregulation of specific ETC subunits in the brain, potentially reflecting early compensation prior to metabolic collapse [151]. These findings highlight the dynamic nature of mitochondrial alterations, which evolve with disease stage and tissue type.

Structural and functional interdependence among ETC complexes adds another layer of complexity. Complexes do not operate in isolation but form higher-order assemblies (supercomplexes), meaning that dysfunction in one component can destabilize others. For instance, complex IV deficiency not only impairs electron transfer but also reduces complex I stability and activity [152]. In AD, direct interactions between Aβ and complex IV exacerbate mitochondrial dysfunction, linking classical pathological hallmarks with bioenergetic failure [140]. In obesity and metabolic disease, reduced complex IV activity is associated with impaired fat oxidation, WAT expansion, and systemic metabolic imbalance, reinforcing a shared mitochondrial phenotype.

Finally, alterations in ATP synthase (complex V) illustrate the convergence of bioenergetic failure and cell fate regulation. Reduced ATP production, oxidative modification of ATP synthase subunits, and its involvement in permeability transition pore formation connect mitochondrial dysfunction to apoptosis and neuronal loss in AD [99]. Similarly, in obesity, impaired ATP synthesis in skeletal muscle contributes to reduced metabolic flexibility. Notably, this phenotype can be partially reversed in individuals responsive to caloric restriction, highlighting the plasticity of mitochondrial function [153]. A detailed description of how individual ETC complexes are affected in AD and obesity, revealing shared patterns of altered ETC enzyme function is provided in Supplementary Table S2.

Collectively, these findings indicate that AD and obesity share a pattern of ETC dysfunction characterized by impaired electron transport, excessive ROS production, compensatory remodeling, and structural interdependence of complexes.

### 5.3. Oxidative Stress in AD and Obesity

Alterations in the ETC, as discussed above, promote excessive production of ROS, thereby linking mitochondrial dysfunction in both AD and obesity to the development of oxidative stress. Oxidative stress arises when ROS and reactive nitrogen species (RNS) exceed endogenous antioxidant defenses.

In the brain, oxidative stress is closely linked to AD pathology. Aβ plaques, composed mainly of Aβ1-40 and Aβ1-42 peptides, form via sequential cleavage of APP by β- and γ-secretases. ROS promote amyloidogenic processing, while Aβ oligomers further impair mitochondria by increasing ROS, inhibiting antioxidant enzymes, and suppressing mitochondrial Aβ-binding alcohol dehydrogenase activity. Transition metals such as Zn, Cu, and Fe amplify oxidative stress via Fenton chemistry. Heme deficiency exacerbates this by promoting APP dimerization and reducing complex IV activity, establishing a self-reinforcing cycle of Aβ overproduction [154]. Tau pathology also begins early in AD; glycogen synthase kinase-3, a major tau-phosphorylating enzyme, becomes activated in oxidizing conditions, and tau, in the presence of ROS, interacts with kinases that promote further phosphorylation and aggregation, linking oxidative stress to tau pathology [155].

Similarly, oxidative stress in adipose tissue drives obesity. Elevated ROS, coupled with increased NADPH oxidase activity and reduced antioxidant defenses, dysregulates adipokine and inflammatory mediator expression [156]. In cultured adipocytes, high fatty acid exposure suppresses adiponectin, whereas ROS scavengers restore adipokine function and improve metabolic parameters [156]. Because ROS levels in adipose tissue are higher than in many other organs, oxidative stress may act as a causal driver rather than a downstream consequence of obesity. Antioxidant interventions can improve adiponectin levels and metabolic outcomes [157].

As stated above, excess ROS damages the TCA cycle enzymes and ETC components, leading to loss of mitochondrial membrane potential, reduced respiratory capacity, and global mitochondrial dysfunction. In obesity, chronic caloric excess overloads the TCA cycle and drives ROS generation at the ETC, whereas in AD, glucose hypometabolism and mitochondrial decline impair these mechanisms. Together, these bioenergetic deficits provide a mechanistic bridge linking systemic metabolic stress with neurodegenerative vulnerability.

## 6. Gut–Brain Axis

Building on the central role of mitochondrial dysfunction and oxidative stress in linking systemic metabolic alterations to neurodegeneration, the gut microbiota has emerged as a key modulator of these processes. By influencing adipose–brain communication, inflammation, and energy metabolism, the gut microbiome may impact mitochondrial function and ROS generation in both central and peripheral tissues.

The human microbiome is a diverse community of microorganisms inhabiting various organs, particularly the gastrointestinal tract. This ecosystem contributes to host health by supporting immune and metabolic systems, nutrient absorption, and gut–brain communication. The interaction between the gut microbiota and the CNS, known as the gut–brain axis, is mediated by endocrine, neural, and immunological signals [158]. Major mechanisms include immune modulation, tryptophan metabolism, vagus nerve activation, engagement of the enteric nervous system, and production of microbial metabolites such as short-chain fatty acids (SCFAs), branched-chain amino acids, peptidoglycans, and secondary bile acids. Through these pathways, the gut microbiota can influence cognitive function, emotional regulation, and stress responses [159].

Microbiota composition is shaped by host genetics, diet, antibiotics, infections, and stress. Although relatively stable in adulthood, aging is associated with reduced microbial diversity [160]. Patterns vary across species; for instance, 24-month-old mice may show increased diversity with age, whereas humans typically show a decline [161].

Intestinal permeability is a critical mediator between the microbiota and cognitive function. Dysbiosis—caused by obesity, stress, or aging—can increase gut permeability. This allows bacterial components such as lipopolysaccharides and metabolites like SCFAs to enter circulation. This promotes systemic and cerebral inflammation, disrupts gut–brain signaling, and increases vulnerability to cognitive decline and AD-related pathology.

SCFAs, particularly acetate, propionate, and butyrate, are produced through fiber fermentation and play key roles in intestinal and neuroimmune homeostasis [162]. They maintain intestinal immune balance and, in small amounts, reach circulation to modulate inflammation and protect against obesity-associated fat accumulation [163]. In AD animal models, SCFAs improve cognition, reduce Aβ deposition, strengthen the blood–CSF barrier, and enhance microglial function [164,165]. Fiber-rich diets, such as the Mediterranean diet, promote bacteria like *Paraprevotella* and *Bacteroides* that produce SCFAs, amino acids, vitamins, and lipids, supporting cognitive and metabolic health. Human studies link these diets to lower dementia risk, independent of genetics [166].

A stable gut microbiota maintains neuroimmune homeostasis, while dysbiosis increases intestinal permeability, promotes inflammation, and disrupts gut–brain signaling [159]. High interindividual variability complicates establishing direct causal links between microbiota changes and neurodegenerative disorders.

Evidence indicates that the microbiota modulates AD pathogenesis. Altered composition is associated with increased gut permeability, systemic and cerebral inflammation, neuronal apoptosis, and Aβ accumulation [159]. Animal studies show that germ-free conditions or microbiota manipulation reduce Aβ deposition and modify microglial function [167]. Interventions, such as probiotics or fecal microbiota transplantation from healthy donors, improve cognition, reduce inflammation, and limit oxidative stress [168]. Conversely, fecal transplantation from AD patients can induce cognitive deficits in healthy models.

Recent findings suggest that microbiota effects extend beyond amyloidosis, influencing tau pathology and neurodegeneration, often in interaction with host genetics, such as ApoE gene variants [169,170]. Human studies associate gut microbiota composition with preclinical neuropathological markers, suggesting potential early risk indicators [170]. Mechanistic evidence comes primarily from germ-free animals, antibiotics, and fecal transplantation, which demonstrate microbiota impacts on neuronal development, neurotransmission, and immune regulation [171,172]. In humans, interventions with probiotics, prebiotics, synbiotics, postbiotics, or fecal transplantation show potential benefits, though clinical results vary. For example, oligosaccharides from brown algae can reshape gut microbiota, reduce CNS immune cell infiltration, attenuate neuroinflammation, and improve behavioral deficits in experimental models [159,171].

As noted previously, obesity contributes to chronic low-grade inflammation, partly via hypertrophic adipocytes with altered adipokine secretion, increasing cognitive decline risk. It is also associated with gut dysbiosis, including reduced microbial diversity and altered taxa, which may compromise intestinal barrier integrity and modulate the gut–brain axis [173,174,175,176]. These changes contribute to metabolic consequences that favor AD-related neuropathology, including Aβ deposition, microglial dysfunction, and neuroinflammation [173,174].

In humans, AD patients exhibit altered gut microbiota, including changes in *Firmicutes*, *Bacteroidetes*, and *Bifidobacterium*, some correlating with Aβ and phosphorylated tau burden. Interindividual differences driven by diet, geography, genetics, and methodological variability complicate identifying universal dysbiosis patterns. Research is now shifting toward functional microbiome characterization, emphasizing metabolic capacity, bioactive metabolite production, and immune interactions [169,177].

Overall, the evidence indicates that the microbiota and its metabolites may act as key modulators of AD pathophysiology. Obesity-related dysbiosis promotes a pro-inflammatory state that disrupts protective metabolic signaling, increasing the risk of cognitive decline and neurodegeneration, particularly in individuals with reduced metabolic or cognitive resilience.

## 7. Conclusions

Based on the evidence presented in this review, AD and obesity share convergent metabolic disturbances that often emerge early, preceding overt clinical manifestations. Key shared mechanisms include mitochondrial dysfunction, with coordinated impairments in the TCA cycle and ETC leading to reduce ATP production and excessive ROS generation; oxidative stress, which damages macromolecules and promotes pathological cascades such as Aβ aggregation and tau phosphorylation in the brain; dysregulation of adipokine signaling in adipose tissue; and systemic metabolic inflammation, linking peripheral energy imbalance to neurodegenerative vulnerability.

These observations underscore the importance of early metabolic alterations as drivers rather than mere consequences of disease progression. Accordingly, targeting these pathways may offer promising avenues for intervention. Emerging therapeutic strategies include mitochondrial enhancers, insulin-sensitizing agents, modulators of adipokine signaling, antioxidants, and interventions aimed at restoring gut–brain axis homeostasis. Such approaches may serve as preventive or disease-modifying measures, potentially delaying or mitigating both neurodegenerative and metabolic disturbances.

Overall, understanding these intersecting mechanisms highlights the shared bioenergetic and inflammatory vulnerabilities of AD and obesity. This integrated framework connects mechanistic insights with potential interventions, enhancing our understanding of disease progression and supporting translational research for targeted therapies.

## Data Availability

No new data were created or analyzed in this study.

## Supplementary Materials

The following supporting information can be downloaded at: https://www.mdpi.com/article/10.3390/cells15080672/s1, Table S1: TCA cycle enzyme alterations in AD and obesity: sheared metabolic vulnerability. Table S2: Mitochondrial electron transport chain complex alterations in AD and obesity.

## Abbreviations

AAV	adeno-associated virus
AD	Alzheimer’s disease
APOE	apolipoprotein E
APP	amyloid precursor protein
Aβ	amyloid β
BACE1	β-site APP cleaving enzyme 1
BAT	brown adipose tissue
BBB	blood–brain barrier
BMI	body mass index
CNS	central nervous system
CSF	cerebrospinal fluid
CVOs	circumventricular organs
DAG	diacylglycerol
DHA	docosahexaenoic acid
ETC	electron transport chain
EV	extracellular vesicle
FH	Fumarase
HDL	high-density lipoprotein
HFD	high-fat diet
IDH	isocitrate dehydrogenase
LDL	low-density lipoprotein
MCI	mild cognitive impairment
MDH	malate dehydrogenase
MHO	metabolically healthy obesity
NPD1	neuroprotectin D1
PC	phosphatidylcholine
PE	phosphatidylethanolamine
PI	phosphatidylinositol
PLC	phospholipase C
PLD	phospholipase D
POMC	proopiomelanocortin
ROS	reactive oxygen species
SAT	subcutaneous adipose tissue
SCFAs	short-chain fatty acids
SDH	succinate dehydrogenase
TCA	tricarboxylic acid
VAT	visceral adipose tissue
VLDL	very-low-density lipoprotein
WAT	white adipose tissue
α-KG	α-ketoglutarate
α-KGDH	α-ketoglutarate dehydrogenase
