# Supplementary material for: From Lipids to Mitochondria: Shared Metabolic Alterations in Obesity and Alzheimer’s Disease
Source: Cells. 2026 Apr 10;15(8):672. doi: 10.3390/cells15080672 (PMC13114639; doi:10.3390/cells15080672)
Supplement: Supplementary file 1 [file cells-15-00672-s001.zip › cells-4222366-supplementary tables.pdf]

Supplementary Table S1

Enzyme (Gene)	TCA Cycle Step	Findings in Alzheimer's Disease	Findings in Obesity
**Citrate Synthase (CS)**	Step 1<br>Acetyl-CoA + OAA → Citrate	↓ Multiple brain regions [109]<br>Citrate reduces Aβ aggregation [110] & ↓ApoE4 carriers [111]<br>↓Plateletsin AD patients [112]	↓ High-fat diet & obese patients [113, 114]<br>Mixed activity in mice strains likely due to single nucleotide polymorphism [115]
**Aconitase 2**	Step 2<br>Citrate ⇄ Isocitrate	↓ peripheral lymphocytes (MCI/AD) [116]<br>Neutral in postmortem AD brains [117]	↑ activity ↑ adipogenesis, ATP production [119]<br>↑ activity (60% increase on HFD) promotes β-oxidation [120]
**Isocitrate Dehydrogenases (IDH1/2/3)**	Step 3<br>Isocitrate → α-KG	↑ IDH1,↓ IDH2 and IDH3 in AD brain transcriptomics [123]	↓ IDH1/2 with Metabolic Syndrome progression [121]
**α-Ketoglutarate Dehydrogenase**	Step 4 (rate-limiting)<br>α-KG → Succinyl-CoA	↓ α-KG in multiple brain regions<br>↓ Correlates with cognitive decline [117,123]	↓ Impairs BAT adipogenesis<br>α-KG supplementation prevents obesity in aged mice [122]
**Succinyl-CoA Synthetase**	Step 5<br>Succinyl-CoA ⇄ Succinate (ATP/GTP)	Protein succinylation of APP/τ<br>→ Aβ accumulation & τ aggregation [124]	Limited direct data<br>↑succinic acid reduces obesity via WAT browning [126,127]
**Succinate Dehydrogenase (SDH / Complex II)**	Step 6<br>Succinate → Fumarate<br>($FADH_2$ → ETC)	↑ Post-mortem brain [117]<br>$A\beta_{25-35}$ suppress SDH in primary cultured neurons [125]	↓ SDH → ↓ BAT thermogenesis<br>↓ Skeletal muscle (obese patients)<br>Succinate oxidation protects vs. obesity [128]
**Fumarase (FH)**	Step 7<br>Fumarate ⇄ Malate	↓ FH in systematic analyses [123]<br>(earlier studies: neutral) [117]	↑glucose ↑ fumarate→ metabolic syndrome [130]<br>FH deficiency → protection from obesity & insulin resistance [131]
**Malate Dehydrogenase (MDH1/2)**	Step 8<br>Malate ⇄ OAA<br>(NADH yield)	↑ Post-mortem brain [117]<br>↓ peripheral blood cells from AD patients [123]; ↑ CSF(candidate biomarker) [129]	↓ MDH2 in CSF after bariatric surgery → improved glycemia [132]

Supplementary Table S1: TCA cycle enzyme alterations in AD and Obesity: shared metabolic vulnerability

Supplementary Table S2

ETC Complex (Protein)	Primary Function	AD Findings	Obesity Findings
**Complex I (NADH Dehydrogenase)**	NADH → Ubiquinone<br>Proton pumping<br>Major ROS site	↓ Expression & activity in brain<br>↓ ATP production<br>Early decline on PET imaging [137]<br>↑ Platelets [150]	Complex I subunit KO → ↑ obesity & inflammation [144]<br>↑ Complex I subunits, NDUFS3/NDUFS9 on Western diet (pigs) [151]
**Complex II (Succinate Dehydrogenase)**	Succinate → Fumarate<br>Electron entry into ETC<br>No proton pumping	See TCA enzyme findings (Table I)	See TCA enzyme findings (Table I)
**Complex III (Cytochrome bc_1)**	Ubiquinol → Cytochrome c<br>Major ROS source (Q-cycle)	↓ Activity in AD brain [147]<br>Inhibiting Complex III ROS → ↓ plaques [148]	ROS from Complex III required for adipocyte differentiation [149]
**Complex IV (Cytochrome c Oxidase)**	Electrons → O_2<br>Proton pumping<br>Terminal oxidase	↓ All cortical regions [139]<br>Inhibited by Aβ peptides in the presence of copper [140]↓ MCI platelets (early biomarker) [141]	↓ With aging → WAT expansion [145]
**Complex V (ATP Synthase)**	ATP generation from proton gradient (F_0F_1-ATPase)	↓ Activity & oxidative modification<br>Altered α/β subunits [99]	↓ ATP synthase in obese muscle [143]

Supplementary Table S2. Mitochondrial electron transport chain complex alterations in AD and obesity
